# TFIIB–Termination Factor Interaction Affects Termination of Transcription on Genome-Wide Scale

**DOI:** 10.3390/ijms25168643

**Published:** 2024-08-08

**Authors:** Michael J. O’Brien, Jared M. Schrader, Athar Ansari

**Affiliations:** Department of Biological Science, 5047 Gullen Mall, Wayne State University, Detroit, MI 48202, USA; mjobrien@wayne.edu (M.J.O.); schrader@wayne.edu (J.M.S.)

**Keywords:** budding yeast, genome-wide analyses, RNA polymerase II, TFIIB, transcription

## Abstract

Apart from its well-established role in the initiation of transcription, the general transcription factor TFIIB has been implicated in the termination step as well. The ubiquity of TFIIB involvement in termination as well as mechanistic details of its termination function, however, remain largely unexplored. Using GRO-seq analyses, we compared the terminator readthrough phenotype in the *sua7-1* mutant (TFIIB*^sua7-1^*) and the isogenic wild type (TFIIB*^WT^*) strains. Approximately 74% of genes analyzed exhibited a 2-3-fold increase in readthrough of the poly(A)-termination signal in the TFIIB*^sua7-1^* mutant compared to TFIIB*^WT^* cells. To understand the mechanistic basis of TFIIB’s role in termination, we performed the mass spectrometry of TFIIB—affinity purified from chromatin and soluble cellular fractions—from TFIIB*^sua7-1^* and TFIIB*^WT^* cells. TFIIB purified from the chromatin fraction of TFIIB*^WT^* cells exhibited significant enrichment of CF1A and Rat1 termination complexes. There was, however, a drastic decrease in TFIIB interaction with CF1A and Rat1 complexes in the TFIIB*^sua7-1^* mutant. ChIP assays revealed about a 90% decline in the recruitment of termination factors in the TFIIB*^sua7-1^* mutant compared to wild type cells. The overall conclusion of these results is that TFIIB affects the termination of transcription on a genome-wide scale, and the TFIIB–termination factor interaction plays a crucial role in the process.

## 1. Introduction

The RNAPII transcription cycle consists of initiation, elongation, and termination steps. Accomplishing each step requires a set of accessory factors. The conventional view is that the factors operating at each step have distinct, independent roles specific to that step. However, a large body of genetic, biochemical, and functional evidence from yeast and higher eukaryotes has challenged this dogma. It has been demonstrated that there is extensive crosstalk between the promoter and terminator-bound factors during the transcription cycle [1,2,3,4,5,6,7]. Recent studies have revealed that at least some initiation factors are involved in the termination step of transcription [8]. Proper termination is vital for the production and nucleocytoplasmic transport of mature mRNA transcripts [9,10,11,12]. The identification of general transcription factors involved in the termination is, therefore, crucial for a thorough understanding of the transcription cycle and eukaryotic gene expression.

TFIIB is a general transcription factor (GTF) and an essential component of the preinitiation complex (PIC). During the initiation of transcription, TFIIB performs a critical role in promoter recognition, RNAPII recruitment, and transcription start site selection [13,14]. It binds directly to sequences in the promoter region and makes multiple contacts with the components of the PIC [15,16,17,18]. Genome-wide analyses employing ChIP-Seq demonstrated TFIIB occupancy in the 5′ end of genes [18,19,20] in accordance with its established role in the initiation of transcription. It was, however, surprising to find TFIIB occupying the 3′ end of many genes in budding yeast [2,21,22,23,24,25]. At least in some genes, the TFIIB ChIP signal at the 3′ end may be due to the presence of an anti-sense promoter there [26]. Another study using the ChIP-exo approach, however, failed to identify TFIIB at the 3′ end of yeast genes [17]. The ChIP-exo approach involves the digestion of immunoprecipitated chromatin with λ exonuclease, which digests the DNA except the region protected by the bound protein. The lack of 3′ TFIIB occupancy by ChIP-exo, therefore, suggests TFIIB does not directly bind to the DNA near the 3′ end.

The ChIP-exo results suggest that TFIIB crosslinks to the 3′ end of genes due to protein–protein interactions. This view is supported by the discovery of multiple interactions of TFIIB with the factors operating at the 3′ end of genes [8]. One of the first and noteworthy interactions was with Ssu72, which is a subunit of the CPF 3′ end processing–termination complex. TFIIB exhibits both a genetic and physical interaction with Ssu72 [27,28,29,30]. Further investigation revealed the interaction of TFIIB with yeast CF1 termination complex subunit Rna15 as well as its human homolog CstF64 [23,31]. The yeast study culminated in the purification of a complex of TFIIB with Rna14, Rna15, Pcf11, Clp1, and Hrp1 subunits of the CF1 complex [2]. In addition to the CF1 complex, TFIIB was shown to interact with a number of subunits of the CPF 3′ end processing–termination complex [32]. Recently, we demonstrated that the interaction of TFIIB with all three yeast termination complexes takes place almost exclusively in the context of chromatin [33]. Taken together, these studies strongly suggested that TFIIB may have a hitherto unidentified role in the termination step of transcription.

Although most of the TFIIB–termination factor interactions were observed in yeast, the first report of the factor playing a role in termination came from a mammalian study [31]. This study also found that TFIIB phosphorylation at serine-65 is necessary for its termination function. TFIIB serine-65 phosphorylation regulates its interaction with the Cstf-64 subunit of the CstF termination complex and facilitates recruitment of the complex at the 3′ end of a gene [31]. In budding yeast, TFIIB’s involvement in termination was demonstrated using the *sua7-1* mutant, which has glutamic acid replaced by lysine at the 62nd position [30]. In this mutant, the promoter recruitment of TFIIB remains unaffected, but its terminator occupancy is diminished [21]. Consequently, the recruitment of termination factors at the 3′ end of genes is compromised in the *sua7-1* mutant, leading to a termination defect [30]. TFIIB also affects poly(A)-independent termination, which is an alternate pathway for the termination of transcription unique to budding yeast [34]. The role of TFIIB in poly(A)-dependent termination was demonstrated in flies as well [35].

TFIIB has been implicated in the termination of transcription of just a few genes in budding yeast and humans and only one gene in flies. The global role of TFIIB in termination, however, remains largely unexplored [30,31,35]. The biochemical basis of TFIIB’s contribution to termination is also unclear. Understanding the prevalence of TFIIB involvement in termination and the mechanistic basis of this function is crucial for understanding its comprehensive role in the transcription cycle. We, therefore, performed GRO-seq analysis and mass spectrometry of affinity-purified TFIIB from the wild type and the *sua7-1* mutant. Our results demonstrate that TFIIB–termination factor interaction affects the termination of transcription on a genome-wide scale in budding yeast.

## 2. Results

### 2.1. TFIIB Affects Termination of Transcription on a Genome-Wide Scale

To determine the role of TFIIB in termination, we used the *sua7-1* mutant of TFIIB (TFIIB*^sua7-1^*). This mutant has glutamic acid replaced by lysine at position 62 (E62K) [27]. The mutant is cold-sensitive and exhibits altered transcription start site selection [28]. The binding affinity of TFIIB*^sua7-1^* for the promoter, as well as its interactions with TBP and RNAPII, are comparable to that of wild type TFIIB (TFIIB*^WT^*) [36]. The recruitment of general transcription factors and RNAPII onto the promoter during the assembly of the preinitiation complex is also indistinguishable from wild type [36]. TFIIB has been observed to crosslink to the 3′ end of genes in TFIIB*^WT^* cells [21]. In TFIIB*^sua7-1^*, however, the 3′ end crosslinking is lost, and consequently, promoter–terminator crosstalk or gene looping is diminished as well (Singh and Hampsey, 2007). Therefore, the TFIIB*^sua7-1^* mutant is a suitable target for investigating a termination defect at the 3′ end of genes.

**Figure 1:**
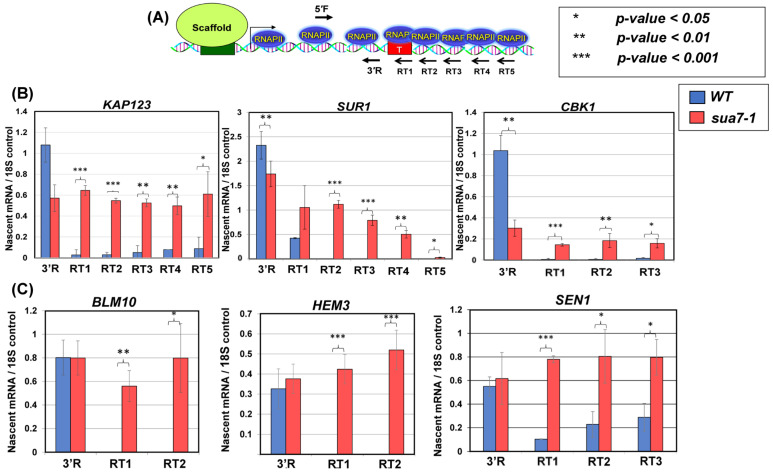
**TRO shows a transcription termination defect for six genes in the sua7-1 mutant:** (**A**) Schematic depiction of a gene showing the actively transcribing RNA polymerase II and positions of primers used for cDNA synthesis following TRO procedure. Primers are depicted with arrows; primers within the gene body are primers 5′F and 3′R, whereas primers RT1-5 are downstream of the terminator region. (**B**,**C**) Quantification of RNA levels detected following TRO analysis in in wild type and sua7-1 mutant cells. Results shown here are from three biological replicates. RT-PCR signal is represented as nascent mRNA signal compared to 18S control. RT reactions performed with individual primers, as shown in diagram. PCR reaction performed with primer pair 5′F/3′R. *p*-values are a result of a standard *t*-test. Error bars represent standard deviation.

To determine the role of TFIIB in termination, we performed ‘Transcription Run-On’ (TRO) assays in TFIIB*^sua7-1^* and TFIIB*^WT^* cells for *BLM10*, *HEM3*, *SEN1*, *CBK1*, *KAP123* and *SUR1* genes. These genes were selected because their next immediate downstream gene is at least 500 bp away, and therefore, any termination defect can be determined with confidence. The TRO assay detects the presence of transcriptionally active polymerases on a gene. When termination is defective, the TRO signal is detected downstream of the poly(A) termination site (Figure 1A). Strand-specific TRO analysis revealed that in all six genes, there was a strong polymerase signal in the coding region before the poly(A) site in wild type cells (Figure 1B,C, blue bars). There was, however, a strongly reduced or undetectable polymerase signal in the region downstream of the poly(A) site (Figure 1B,C, blue bars). In TFIIB*^sua7-1^* cells, though there was no difference in the TRO signal upstream of the poly(A) site compared to the wild type, there was a dramatic increase in TRO signal in the downstream regions of all six genes (Figure 1B,C, red bars). This revealed a failure to read the termination signals efficiently in the TFIIB*^sua7-1^* mutant and a continued transcription of downstream regions. The termination defect in TFIIB*^sua7-1^* cells is not due to an indirect effect of mutation on termination factor levels as the Western blot signal of Rna15 and Pta1 is similar in the wild type and TFIIB*^sua7-1^* cells (Supplementary Figure S1).

**Figure 2:**
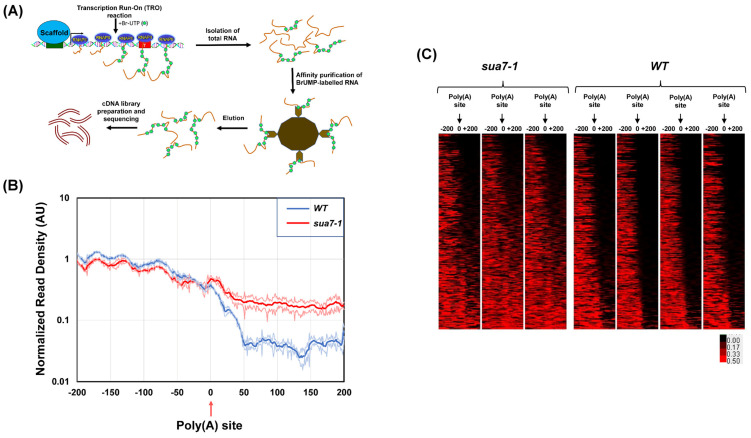
**GRO-seq demonstrates a genome-wide defect in transcription termination in the sua7-1 mutant:** (**A**) Schematic showing the major steps of the GRO-seq approach, culminating in a sample prepared for sequencing. (**B**) Metagene plot of normalized read densities. WT and sua7-1 normalized read densities are plotted from −200 nt upstream of poly(A) site to +200 nt downstream of poly(A) site. Thick/center lines are the average normalized read densities. Upper and lower (light-colored) lines represent standard error. Data are from three biological replicates. (**C**) Heat map shows GRO-seq signal of the same set of 337 genes from −200 nt upstream of poly(A) site to +200 nt downstream of poly(A) site in WT and sua7-1 cells. All three replicates are shown here. The genes are arranged from top to bottom in order of increasing terminator readthrough phenotype in TFIIB^sua7-1^ cells. For each gene, the GRO-seq signal in TFIIB^sua7-1^ and TFIIB^WT^ cells is indicated according to the red/black scale.

These results prompted us to examine TFIIB’s involvement in termination on a genome-wide scale. To accomplish this, we used the ‘Global Run-On-Seq’ (GRO-seq) approach, which is the genome-wide version of the strand-specific TRO approach [37]. Briefly, GRO-seq involves the incorporation of BrUTP in newly synthesized RNA, affinity purification of nascent labeled RNA, reverse transcription, and high throughput sequencing of the cDNA library, as shown in Figure 2A. GRO-seq provides a high-resolution snapshot of the position and density of actively transcribing polymerase in a strand-specific manner (Figure 2B). GRO-seq was performed with TFIIB*^sua7-1^* and TFIIB*^WT^* strains with three biological replicates. GRO-seq samples were first stripped of adapter sequences using cutadapt. The 3′ end reads were then aligned to the yeast s288c genome downloaded from SGD (version R64-3-1). Throughout this analysis, we used the 3′ annotated end of mRNAs from SGD for cells grown in YPD media corresponding to the poly(A) cleavage site. We restricted our analysis to 2501 protein-coding genes whose 3′ ends were at least 500 bp away from the neighboring gene on the same strand. This was done because of the compact nature of the yeast genome, which often results in the terminator region of a gene overlapping with the promoter or terminator elements of the neighboring gene. To restrict our analysis to transcriptionally active genes in YPD growth conditions, we selected mRNAs that were at least 500 nucleotides in length or longer and whose expression value was more than or equal to 1 read/nucleotide within the CDS in at least three biological replicates. We, therefore, performed our final analysis with 337 genes that satisfied all benchmarks described above. To avoid the results being skewed in favor of long and highly expressed genes, we normalized by dividing the number of reads by the length of the gene.

To compare the termination of transcription in TFIIB*^sua7-1^* and TFIIB*^WT^* cells, GRO-seq reads were aligned in a 200 bp window upstream and 200 bp downstream of the poly(A) site. A metagene plot shows both TFIIB*^WT^* and TFIIB*^sua7-1^* exhibiting a similar GRO-seq profile in the region upstream of the poly(A) site (Figure 2B). There was, however, a sharp drop in the number of reads beyond the poly(A) site in TFIIB*^WT^* cells (Figure 2B, blue line). In TFIIB*^sua7-1^*, there was also a decrease in the number of reads after the poly(A) site, but the number of reads was higher than in TFIIB*^WT^* (Figure 2B, red line). Heat maps of individual replicates demonstrate that there is hardly any detectable GRO-seq signal beyond the poly(A) site in TFIIB*^WT^* cells in all replicates (Figure 2C). In the TFIIB*^sua7-1^* mutant, however, nearly 70% of genes exhibited a varying degree of GRO-seq signal in the region downstream of the poly(A) site in all three replicates (Figure 2C). Genes in the bottom 40% section of the heat map show strong GRO-seq signals, while the upper 25% of genes show no GRO-seq signal after the poly(A) site in the mutant (Figure 2C). These results indicate that, in nearly two-thirds of the analyzed genes, the polymerase was unable to read the poly(A) termination signal efficiently in TFIIB*^sua7-1^* cells, leading to an enhanced readthrough in the region downstream of the poly(A) site (Figure 2C).

**Figure 3:**
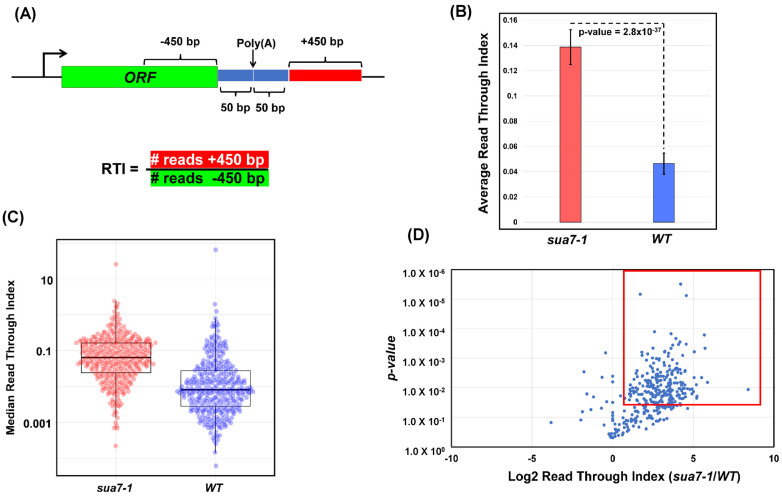
**A majority of genes have an increased Read Through Index in sua7-1:** (**A**) Schematic representing calculation of Read Through Index (RTI) for individual genes. RTI is calculated from number of reads +450 bp divided by number of reads −450 bp. (**B**) The average Read Through Index of all analyzed genes in sua7-1 and WT cells. Data represented are from three biological replicates, and *p*-value was obtained from standard 2-sided, paired *t*-test. (**C**) Median Read Through Index for all analyzed genes in sua7-1 and WT cells represented as a box and whisker plot. The middle line represents the median value of the corresponding RTI. The top and bottom lines represent the upper and lower quartiles. Data are represented from three biological replicates and *p*-value obtained from standard 2-sided *t*-test with unequal variance. n = 337 is the total number of analyzed genes. (**D**) The log2 RTI ratio of sua7-1/WT is plotted on the *x*-axis with *p*-value plotted on the *y*-axis. All genes analyzed are plotted as blue dots. The outlined red window contains all genes that display at least a 2-fold increase in RTI ratio of sua7-1/WT while simultaneously having a *p*-value less than 0.05. Data are represented from three biological replicates and *p*-value obtained from standard 1-sided *t*-test with unequal variance.

To further characterize the role of TFIIB in termination on a genome-wide scale, we calculated the readthrough index (RTI) in the mutant and wild type cells as described in [38]. The RTI was calculated by measuring the ratio between the number of read counts 500 bp downstream and 500 bp upstream of the poly(A) site, excluding a region of 50 bp directly up- and downstream of the poly(A) site, as shown in Figure 3A. A termination defect is expected to result in more GRO-seq reads downstream of the poly(A) site compared to the upstream region and, therefore, a higher RTI value.

The RTI value in the mutant (RTI = 0.139) registered a statistically significant 3-fold increase over the wild type cells (RTI = 0.046) (Figure 3B). These results suggest that TFIIB affects the termination of transcription on a genome-wide scale in budding yeast. The RTI values in Figure 3B represent mean values, which are a good indicator of the trend if data follow a symmetric distribution. The mean values, however, can be skewed by outliers. In contrast, median values are not affected by outliers and more accurately represent the general trend. Therefore, to further probe if termination in the mutant is affected on a genome-wide scale, median RTI values of genes in the mutant and the wild type strains for all replicates were charted in a box and whisker plot. Median RTI values calculated from this plot are 0.063 and 0.008 for TFIIB*^sua7-1^* and TFIIB*^WT^*, respectively (Figure 3C), making the median RTI > 7-fold higher than the wild type. These results corroborate the conclusion drawn from mean RTI values shown in Figure 3B that TFIIB affects the termination of transcription on a genome-wide scale.

We next determined what fraction of genes used in this analysis display defective termination in TFIIB*^sua7-1^*. The log2 values of RTI ratios of TFIIB*^sua7-1^*/TFIIB*^WT^* for all genes were plotted as a function of *p*-values (Figure 3D). The boxed area in the plot represents all genes that exhibit a log2 RTI ratio of more than one, indicative of a 2-fold increase in the RTI of TFIIB*^sua7-1^* compared to TFIIB*^WT^* and a *p*-value of less than 0.05. Based on these criteria, nearly 73.8% of genes analyzed in this study show a statistically significant dependence on TFIIB for efficient termination of their transcription. This is in contrast to Pcf11 and Ysh1, which affect the termination of more than 90% of genes in yeast [38]. Thus, TFIIB is not an essential termination factor like CPF and CF1 3′ end processing–termination complexes but has a significant effect on termination of nearly three-fourths of genes used in our analysis.

Next, we searched for common features among genes that conferred dependence on TFIIB for efficient termination of transcription. Genes whose termination is affected by TFIIB do not display common predicted structures or enriched sequence motifs. TFIIB-linked termination is also not related to the length of genes (Supplementary Figure S2A). Furthermore, the TFIIB-associated termination phenotype could not be linked to the presence or absence of TATA-box in the promoter of the gene (Supplementary Figure S2B). Both TATA and TATA-less promoter-containing genes exhibited similar RTI value distribution in the TFIIB*^sua7-1^* strain (Supplementary Figure S2B). Ontological analysis revealed that TFIIB termination dependence was more pronounced in genes associated with translation and ribosomal function, which is not surprising as the fraction analyzed here was already enriched in genes associated with ribosomal function (Supplementary Figure S2C). Thus, TFIIB’s role in termination cannot be attributed to a particular functional category of genes. 

### 2.2. TFIIB’s Interaction with Termination Factors Is Diminished in the Termination Defective Mutant

**Figure 4:**
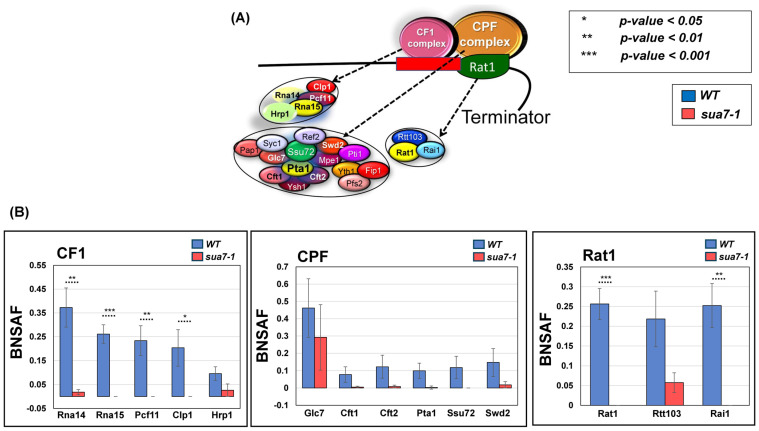
**TFIIB–termination factor interactions are compromised in sua7-1 mutant.** (**A**) Diagram of the three major termination complexes in *S. cerevisiae* and their corresponding subunits. (**B**) BNSAF values of termination factors, grouped by corresponding complexes in sua7-1 and WT cells. Data are from 4 biological replicates. *p*-values obtained from a standard, paired *t*-test. Error bars represent one unit of standard error based on four biological replicates.

Having established the role of TFIIB in the termination of transcription of nearly three-fourths of analyzed genes in budding yeast, we next investigated the mechanism underlying the role of the factor in termination. We hypothesized that TFIIB may affect termination directly by interacting with the termination factors and regulating their activity at the 3′ end of genes or indirectly by affecting CTD-serine-2 phosphorylation, which in turn facilitates the recruitment of termination factors. Our recently published results favored the first possibility [33]. We demonstrated the interaction of TFIIB with all three termination complexes of yeast, CPF, CF1, and Rat1, in the TFIIB*^WT^* strain. The interaction with CF1 and Rat1 complexes was observed exclusively in the chromatin context, thereby suggesting that the interaction occurred only during transcription. We reasoned that if the TFIIB–termination factor interaction is linked to a TFIIB-mediated effect on termination, then the interaction of TFIIB with the termination factors will be reduced in the termination defective TFIIB*^sua7-1^* strain. We, therefore, affinity purified TFIIB from TFIIB*^sua7-1^* cells. Purification was performed from both chromatin and soluble fractions, as described in [33]. Affinity-purified fractions were subjected to mass spectrometry. A quantitative proteomic approach was followed to analyze mass spectrometry data [39,40]. The relative abundance of a termination factor in a purified TFIIB sample was quantified by dividing the SAF value of the factor by that of TFIIB to obtain the TFIIB-normalized spectral abundance factor (BNSAF). The data presented here are the result of four independent replicates. Comparison of the BNSAF values revealed that the interaction of TFIIB with Rna14, Rna15, Pcf11, and Clp1 subunits of CF1 complex as well as Rat1 and Rai1 subunits of Rat1 complex exhibited a statistically significant decline of about 70–90% in TFIIB*^sua7-1^* compared to TFIIB*^WT^* cells (Figure 4B). Interaction with subunits of the CPF complex also registered a drop in the mutant, but it was not statistically significant due to higher sample-to-sample variability (Figure 4B). We next checked if the interaction of TFIIB with GTFs is similarly negatively impacted in the mutant. Contrary to the expectation, TFIIB interaction with subunits of TFIID, TFIIA, and TFIIF exhibited an increase in TFIIB*^sua7-1^* compared to TFIIB*^WT^* cells, but the difference in BNSAF value between TFIIB*^sua7-1^* and TFIIB*^WT^* cells was statistically not significant (Supplementary Figure S3). The termination defective TFIIB*^sua7-1^* mutant, therefore, exhibits a selective diminution in interaction with termination factors. These results suggest that TFIIB either helps in the recruitment of termination factors or stabilizes their recruitment at the 3′ end of genes.

### 2.3. Recruitment of Termination Factors at the 3′ End of Genes Is Compromised in the Termination Defective Mutant

**Figure 5:**
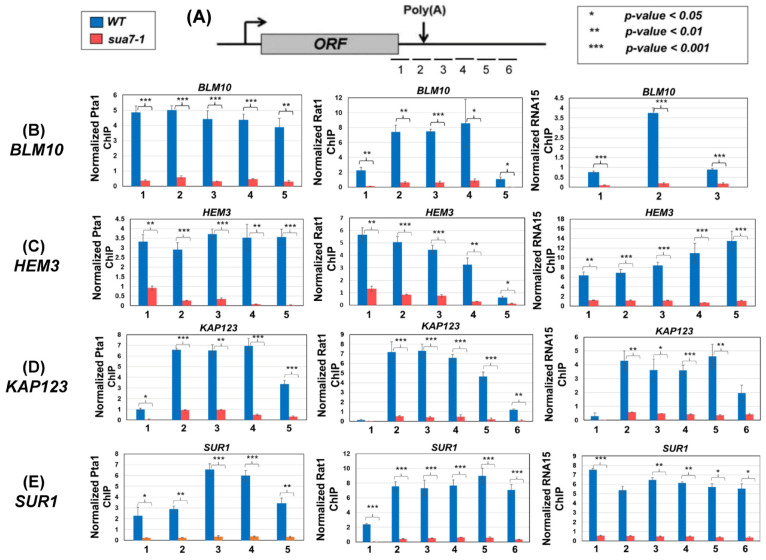
**Termination factor occupancy is reduced at the 3′ end of genes in sua7-1 mutant.** (**A**) Schematic depicting the primer locations for examining occupancy of termination factors on the terminator region and regions directly flanking the terminator in reference to a gene/ORF. (**B**–**E**) ChIP signal for each factor was normalized with the input signal and then with the RNAPII signal in the region. ChIP signal for Pta1, Rat1, and Rna15 at and around the terminator region is significantly decreased in sua7-1 compared to wild type cells for the genes *BLM10, HEM3, KAP123,* and *SUR1*. *p*-values were calculated by standard two-tailed *t*-test. Error bars represent one unit of standard error based on three biological replicates.

To further probe the role of the TFIIB–termination factor interaction in termination, we checked the recruitment of all three termination complexes at the 3′ end of *BLM10*, *HEM3*, *KAP123,* and *SUR1*. These four genes were selected because their next neighboring gene on the same strand is more than 500 bp away, and they exhibit a termination defect in TFIIB*^sua7-1^* cells (Figure 1). The recruitment of the CPF complex was monitored in terms of its Pta1 subunit, the CF1 complex in terms of its Rna15 subunit, and the Rat1 complex using its Rat1 subunit. All three subunits were tagged at their C-terminus by an HA-epitope, and their recruitment at the 3′ end of *BLM10*, *HEM3*, *KAP123,* and *SUR1* was examined in TFIIB*^sua7-1^* and TFIIB*^WT^* cells employing the ChIP approach. The ChIP signal for each factor was normalized with the input signal and then with the RNAPII signal at the site. As expected, all three factors, Pta1, Rna15, and Rat1, were localized at the 3′ end of genes in TFIIB*^WT^* cells (Figure 5B–D, blue bars). In TFIIB*^sua7-1^* cells, however, the recruitment of all three factors registered a drop of nearly 90% (Figure 5B–D, red bars). The decrease in 3′ end crosslinking was statistically significant as the observed *p*-value in every case was less than 0.05 (Figure 5B–D). A possible interpretation of these results is that TFIIB is required for either the recruitment of the termination factors or the stabilization of their association with the 3′ end of genes, or both. The possibility of TFIIB enhancing the exoribonuclease activity of Rat1, thereby expediting the dissociation of the elongating polymerase from the template, however, still cannot be ruled out.

## 3. Discussion

The role of the initiation step in the transcription of a gene is well established. It is generally, however, not appreciated that the termination step is just as vital for the efficient transcription of a gene. The generally accepted view is that transcription termination in budding yeast requires three multiprotein complexes: CF1, CPF, and Rat1 [10,38]. We show here that the general transcription factor TFIIB is also involved in the termination of transcription on a genome-wide scale in budding yeast. Our conclusion is based on four crucial observations. First, TFIIB crosslinks to the 3′ end of a number of transcriptionally active genes in wild type cells but not in the TFIIB*^sua7-1^* mutant [21]. Second, RNAPII reads through the termination signal in the TFIIB*^sua7-1^* mutant. The mutant exhibits a statistically significant increase of two-fold or more in RTI over the TFIIB*^WT^* cells for at least 74% of genes analyzed in our study (Figure 2B). Third, TFIIB interacts with subunits of CF1 and Rat1 3′ end processing–termination complexes in wild type cells. In the termination defective TFIIB*^sua7-1^* mutant, there was a statistically significant reduction in TFIIB interaction with termination factors (Figure 4), suggesting that the TFIIB–termination factor interaction may be crucial for efficient termination. Fourth, recruitment of Pta1, Rna15, and Rat1 termination factors at the 3′ end of genes registered a nearly 90% decline in TFIIB*^sua7-1^* mutant over TFIIB*^WT^* cells (Figure 5). The overall conclusion of these results is that TFIIB is involved in the termination of transcription of nearly three-fourths of genes analyzed in this study in budding yeast.

TFIIB, however, is not an essential termination factor like Pcf11 and Ysh1 subunits of CF1A and CPF complexes, respectively. In the TFIIB*^sua7-1^* mutant, there was no complete loss of termination as was observed upon nuclear depletion of Pcf11 and Ysh1 [38]. Despite the higher GRO-seq signal downstream of the poly(A)-site, the smooth descending polymerase signal was preserved in the mutant. A logical interpretation of these results is that termination still occurs in the mutant, but a significant number of polymerase molecules are unable to recognize the poly(A) signal and read through into the downstream region. These results are in agreement with the termination factors ChIP data that found 3′ end recruitment of all three termination complexes decreasing by about 90% in the TFIIB*^sua7-1^* mutant. A recent genome-wide analysis carried out in mammalian cells also reported polymerases being unable to read the termination signal in the absence of functional TFIIB in the cell [41]. This study performed PRO-seq upon nuclear depletion of TFIIB and found a majority of polymerases reading through the termination signal. The extent of readthrough in the absence of TFIIB in this case was comparable to that observed upon nuclear depletion of Pcf11 and Ysh1 in yeast [38]. It is quite possible that the point mutation in TFIIB*^sua7-1^* affects some aspects of termination, but the mutant TFIIB still retains interactions that allow it to facilitate termination, though with reduced efficiency. It will be interesting to look at the termination phenotype in yeast cells that are depleted of TFIIB in the nucleus by the anchor away approach. These results also demonstrate that the termination function of TFIIB has been conserved during evolution.

It is possible that the termination defect observed in the TFIIB*^sua7-1^* mutant is an indirect effect of the mutation on termination through defective initiation or elongation and not due to the direct involvement of TFIIB in termination. The evidence, however, supports TFIIB’s direct involvement. First, the mutant form of TFIIB is indistinguishable from the wild type counterpart in terms of its ability to initiate transcription in vitro [36]. Second, the GRO-seq profile presented here shows almost identical signals in the open reading frame in TFIIB*^WT^* and TFIIB*^sua7-1^* cells, thereby suggesting that initiation and elongation steps are not adversely affected in the mutant in vivo (Figure 2). Third, TFIIB physically interacts with the termination factors, and this interaction is compromised in the TFIIB*^sua7-1^* mutant (Figure 4). Fourth, TFIIB crosslinks to the 3′ end of genes in the TFIIB*^WT^* cells but not in the termination defective TFIIB*^sua7-1^* mutant [21]. 

TFIIB may affect termination either directly by interacting with termination factors and facilitating or stabilizing their recruitment at the 3′ end of genes or indirectly by influencing CTD-serine-2 phosphorylation. Our results favor a direct role for TFIIB in termination for two reasons. First, TFIIB crosslinks to the 3′ end of genes during transcription. Second, TFIIB exhibits physical interaction with subunits of CF1 and Rat1 termination complexes in the context of chromatin. These interactions are compromised in the termination defective TFIIB*^sua7-1^* mutant. The mammalian study demonstrating the TFIIB termination defect concluded that this defect observed in the absence of TFIIB was an indirect effect of an elongation defect due to excessive incorporation of P-TEFb on chromatin in the absence of TFIIB [41]. However, the study never checked the presence of TFIIB at the 3′ end of genes or the interaction of mammalian TFIIB with termination factors. An independent study, however, revealed that mammalian TFIIB, like its yeast homolog, also interacts with CstF-64, which is the mammalian homolog of yeast Rna15 [31]. Furthermore, TFIIB-CstF-64 interaction was found to be crucial for the termination of transcription in this study. These results corroborate the crucial role of TFIIB–termination factor interaction in the termination of transcription. An independent study demonstrated the role of P-TEFb in regulating the exoribonuclease activity of Xrn2, which is the mammalian counterpart of Rat1, in driving termination [42]. The interaction of TFIIB with Rat1 may similarly stimulate Rat1 activity, leading to efficient termination.

Apart from the termination defect, the *sua7-1* mutant used in this study also exhibits other phenotypes related to transcription. The transcription start site selection is altered in this mutant. The mutant is also defective in gene looping, which is the interaction of the promoter and terminator regions of a gene in a transcription-dependent manner [21]. Previous results from our lab have shown that a gene loop is formed by the interaction of promoter-bound TFIIB with the termination factors occupying the 3′ end of genes [2,23,24]. All gene looping defective mutants that we have analyzed so far in yeast are also defective in termination [2]. Thus, TFIIB’s role in termination may be linked to its ability to facilitate gene loop formation. In fact, TFIIB-mediated gene looping has been found to affect alternative 3′ end processing, which is the selection of transcription termination site at the 3′ end of a gene in budding yeast [4]. A similar role of gene looping in alternative 3′ end processing was recently demonstrated in mammalian cells [5]. These results suggest that the role of TFIIB at the 3′ end of genes is not restricted to facilitating termination but may also involve selection of the transcription termination site from among multiple poly(A) sites present at the 3′ end [8]. Overall, this study serves as a paradigm for studying the broader role of TFIIB in the transcription cycle in higher eukaryotes. The study also opens up avenues for investigating the broader role of factors involved in different steps of the transcription cycle in the overall process of transcription and cotranscriptional RNA processing.

## 4. Materials and Methods

### 4.1. Yeast Strains

Yeast strains (*Saccharomyces cerevisiae*) used in this study are BY4733 with genetic background *MATα his3Δ200 trp1Δ63 leu2Δ0 met15Δ0 ura3Δ0*. All subsequent strains used were derived from BY4733. Supplementary Material S1 lists all strains used in this study along with their genotype.

### 4.2. Protein Purification from Soluble and Chromatin Fractions

Purification of TFIIB from soluble and chromatin fractions was performed exactly as described in [33]. Analysis was performed on four biological replicates, utilizing chromatin and soluble fractions obtained from *S. cerevisiae* strain *sua7-1-HA* (WA158; Supplemental Material S1).

### 4.3. Mass Spectrometry and Quantitative Analysis

Mass spectrometric analysis was performed as described in [33]. Analysis was performed with four biological replicates, utilizing chromatin and soluble fractions obtained from *S. cerevisiae* strain *sua7-1-HA* (WA158; Supplemental Material S1) in conjunction with *wild type* data obtained in O’Brien and Ansari (2024).

### 4.4. GRO-Seq

GRO-seq was performed essentially as described in [43]. Nascent, isolated RNA obtained using GRO-Seq was obtained from three biological replicates of *S. cerevisiae* strains *sua7-1* and *wild type* (WA 304 and *BY4733*; Supplementary Material S1).

### 4.5. GRO-Seq Analysis

GRO-Seq samples were first stripped of adapter sequences using cutadapt. 3′ end reads were then aligned to the yeast s288c genome downloaded from SGD (version R64-3-1). To determine the readthrough index, we used the 3′ end annotations from SGD for mRNAs in YPD media. For each mRNA, we filtered the mRNAs to include only mRNAs whose 3′ ends are at least 500 nucleotides from the next gene on the same strand. Next, we selected mRNAs that were at least 500 nucleotides in length or longer and whose expression values were at least 1 read per nucleotide. We then calculated the readthrough index as the ratio of reads in a downstream window (50 to 500 nucleotides after the 3′ end) divided by an upstream window (−500 to −50 nucleotides in front of the 3′ end) [38]. To determine which mRNAs showed changes in readthrough index in the mutant (*sua7-1*), we compared the replicate readthrough index values from *WT* and mutant and used a 1-sided *t*-test (we ensured readthrough indices were measured in a minimum of five biological samples, with at least two from wild type and at least two in mutant). A criterion of a *p*-value less than 0.05 and a log2 fold change greater than 2 were used to determine which mRNAs showed termination defects.

To generate the metagene plot of the 3′ end, we filtered the mRNAs to include only mRNAs whose 3′ ends are at least 500 nucleotides from the next gene on the same strand. Next, we selected mRNAs that were at least 500 nt in length or longer and whose expression values were at least 1 read/nt. For each mRNA, levels were normalized to the average read density in the CDS. The CDS normalized RNA levels were then averaged and plotted in a window from −200 nt before the 3′ end to +200 nt after the 3′ end. Outside lines represent standard error.

### 4.6. TRO Assay

Transcription Run-On assay was performed essentially as described in [44]. Nascent, isolated RNA gathered using TRO was obtained from three biological replicates of *S. cerevisiae* strains *sua7-1* and *wild type* (WA304 and *BY4733*; Supplementary Material S1). Primers used in TRO are described in Supplementary Material S2.

### 4.7. ChIP

ChIP method and analysis were performed essentially as described in [44]. Analysis was performed with three biological replicates, utilizing DNA obtained from ChIP of *S. cerevisiae* strains (Supplementary Material S1) harboring Pta1-HA (WA167/*wild type* and WA383/*sua7-1*), Rat1-TAP (WA318/*wild type* and WA384/*sua7-1*), and Rna15-TAP WA143/*wild type* and WA386/*sua7-1*). Primers used in TRO are described in Supplementary Material S2.

## Data Availability

The mass spectrometry proteomics data have been deposited to the ProteomeXchange Consortium via the PRIDE partner repository with the dataset identifier PXD041878 and 10.6019/PXD041878. Statistical source data are provided with this article. All other relevant data that support this study are available from the corresponding author upon reasonable request. The GRO-Seq data have been deposited in the NCBI database. The NCBI Geo accession number is GSE259240.

## Supplementary Materials

The following supporting information can be downloaded at https://www.mdpi.com/article/10.3390/ijms25168643/s1.
