# Supplementary material for: TFIIB–Termination Factor Interaction Affects Termination of Transcription on Genome-Wide Scale
Source: Int J Mol Sci. 2024 Aug 8;25(16):8643. doi: 10.3390/ijms25168643 (PMC11354755; doi:10.3390/ijms25168643)
Supplement: Supplementary file 1 [file ijms-25-08643-s001.zip › ijms-3117649-supplementary.pdf]

Supplementary Material

S1 Yeast Strain Table

Strain	Genotype
BY4733	*MATα his3Δ200 trp1Δ63 leu2Δ0 met15Δ0 ura3Δ0*
WA143	*BY4733, MATa his3Δ200 trp1Δ63 leu2Δ0 met15Δ0 ura3Δ0 RNA15-TAP, Histidine* marker
WA147	*MATα his3Δ200 trp1Δ63 leu2Δ0 met15Δ0 ura3Δ0 SUA7-HA::HIS3 PCF11-Myc::TRP1*
WA158	*MATα* cyc1-5000 cyc7-67 ura3-52 leu2-3,112 cyh2 *SUA7-1*-HA (KANMX)
WA167	A HA-His cassette was inserted downstream ORF of *PTA1* in BEK1 strain (*MATα his3Δ200 trp1Δ63 leu2Δ0 met15Δ0 ura3Δ0* KANMX).
WA304	*MATα his3Δ200 trp1Δ63 leu2Δ0 met15Δ0 ura3Δ0 sua7-1*
WA318	*MATα his3Δ200 trp1Δ63 leu2Δ0 met15Δ0 ura3Δ0 RAT1-TAP Tryptophan* marker
WA383	*MATα his3Δ200 trp1Δ63 leu2Δ0 met15Δ0 ura3Δ0 PARENT IS SUA7-1; PTA1-HA , Histidine* marker
WA384	*MATα his3Δ200 trp1Δ63 leu2Δ0 met15Δ0 ura3Δ0 RAT1-TAP Tryptophan* marker, *Sua7-1*
WA386	*MATα his3Δ200 trp1Δ63 leu2Δ0 met15Δ0 ura3Δ0 RNA15-TAP, Tryptophan* marker, *Sua7-1*

S2 Primer Table

Primers used for TRO (RT-PCR):

18S:

Name	Sequence
5’ *18S*	GGAATAATAGAATAGGACGTTGG
3’ *18S*	GTTAAGGTCTCGTTCGTTATCG

BLM10:

Name	Sequence
BLM10 5’F	CCCTATGTTTTTCCCCTAC
BLM10 3’R	TCAGGCATAGTAACTTCTCCA
BLM10 RT1	ATCAGCAGATAGCTCCAG
BLM10 RT2	TGATTTATTACCCGGCAC

CBK1:

Name	Sequence
CBK1 5’F	ACATGGTGGTGCAGATG
CBK1 3’R	AATCTTCCTTGACTGGC
CBK1 RT1	AGAGGAATGTCCTTAACG
CBK1 RT2	AATGTGCGTTCTACATCC
CBK1 RT3	CCCTGATCTGCCATCTC

HEM3:

Name	Sequence
HEM3 5’F	TGTTCCGTTCCTATTGGTG
HEM3 3’R	AGAATTTTCTTTGCGCCAT
HEM3 RT1	ACAACGAAGCTTGTATGAATAG
HEM3 RT2	TTTTTAGAAGTTCACCAAGTG

KAP123:

Name	Sequence
KAP123 5’F	CTGTTCCAGCATTATTGG
KAP123 3’R	GAGCAACAATTCCGTTGT
KAP123 RT1	GTGCCTAAGATTGGGTGG
KAP123 RT2	CACATACATACAAGATTGGC
KAP123 RT3	TCATCGCTGAGTTGATCG

KAP123 RT4	CTTCGAGATGGACGATGC
KAP123 RT5	AATTGCGTCGTCAACATC

SEN1:

Name	Sequence
SEN1 5’F	GCAAATAGGACCCTCTCTC
SEN1 3’R	GGGATAAATGGGCTAGATGA
SEN1 RT1	GGCATTTCTGAAAGTGTAG
SEN1 RT2	ATTTAAAACAGCCCTCGAC
SEN1 RT3	CACTACCTTCTAAACCAATTG

SUR1:

Name	Sequence
SUR1 5’F	GCTTACTACAAGATGCATAG
SUR1 3’R	CCAGTGAGTACTTAGAAG
SUR1 A1	GTTGTGTATTGACGATACTG
SUR1 A2	ACGCAGGATAAGATGTGG
SUR1 RT1	GCAGTATCACTGGTACTT
SUR1 RT2	AGTCTGATATGAATTGTTGG
SUR1 RT3	ACAAGACTGGTCTTCAGG
SUR1 RT4	GTAGTATGTCGTAAGAAGC
SUR1 RT5	GTATAGAGGGTAATATCACAG

Primers used for ChIP:

BLM10:

Pta1: 1:*S/T3R,* ***2:****T1/T3R,* ***3:****T1/T6R,* ***4:****T2/T6,* ***5:****T6/T8*

Rna15: 1:*S/T3R,* ***2:****T1/T3R,* ***3:****T4/T6R*

Rat1: 1:*S/T3R,* ***2:****T1/T6R,* ***3:****T2/T6R,* ***4:****T2/T8R,* ***5:****T4/T6R*

Ser2p: 1:*S/T2R,* ***2:****T2/T6R,* ***3:****T2/T8R,* ***4:****T4/T6R,* ***5:****T6/T8R*

Ser5p 5’ End: 1:*AF/CR,* ***2:****BF/BR,* ***3:****BF/CR,* ***4:****CF/CR*

Ser5p 3’ End: 1:*S/T3R,* ***2:****T1/T3R,* ***3:****T1/ T6R,* ***4:****T2/T6R,* ***5:****T2/ T8R,* ***6:****T4/T6R*

Name	Sequence
BLM10 S	ATATCAGCGTTCCCCTATG
BLM10 T1	ATGGAGAAGTTACTATGCC
BLM10 T2	ACTTGGAAGTTCGATAGAG
BLM10 T4	TAGTGTAACTATGATCACGGC
BLM10 T6	GCATTCTACCTCTAGGGAAG

BLM10 T2R	CTGGAGCTATCTGCTGAT
BLM10 T3R	GTGCCGGGTAATAAATCAA
BLM10 T6R	CTGTATATACACGAGGGCG
BLM10 T8R	GGAAAGAGCTATGACTCTAT
BLM10 AF	GACGATGATATCAAATCACCC
BLM10 BF	GAGAGAAGTCCAGGAAGG
BLM10 CF	ACGCTGGATTATGTTAGTGAC
BLM10 AR	CCTATTGAAGAGTTCTTACCA
BLM10 CR	GACTTACTTGGTAACGATATTGCT

HEM3:

Pta1:* 1:MF/MR,* **2:***5F/3R,* **3:***5F/4R,* **4:***7F/7R,* **5:***7F/8R*

Rna15:* 1:MF/MR,* **2:***5F/4R,* **3:***5F/4AR,* **4:***6F/8R,* **5:***7F/6R*

Rat1:* 1:MF/MR,* **2:***5F/4R,* **3:***5F/4AR,* **4:***6F/4R,* **5:***6F/5R*

Ser2p:* 1:M/FMR,* **2:***7F/8R,* **3:***8F/6R,* **4:***8F/8R,* **5:***8F/9R*

Ser5p 5' End:* 1:A/AR,* **2:***A/BR,* **3:***A/CR,* **4:***B/AR*

Ser5p 3' End:* 1:MF/MR,* **2:***6F/8R,* **3:***7F/8R,* **4:***7F/9R,* **5:***8F/6R*

Name	Sequence
HEM3 MF	AGAAAGGGTGACACCAAGATG
HEM3 5F	CACCATTAATTTGATCAGTCC
HEM3 6F	CCTTATCTCCTTTATCTTTCAGT
HEM3 7F	GACCATTTTCAAATGACCAC
HEM3 8F	ACTATTCATACAAGCTTCGTTG
HEM3 MR	GTTGACGTTGAAGGCACAGAA
HEM3 3R	CCCTCTCATTCCTCTCAT
HEM 4R	CAAATGACCACTGCATAAATAT
HEM 4AR	GAATATGTAATTCCTACCTTTG
HEM3 5R	CTATTCATACAAGCTTCGTTGT
HEM3 6R	CACTTGGTGAACTTCTAAAAA
HEM3 8R	GATCAGAATTCCGACTCCAG
HEM3 9R	CTATCTCTGCCACTCACTAT
HEM3 AF	GGTGGGAGAAAATCGAAATTG
HEM3 BF	CTGATCGAAGAAAAGTATCCG
HEM3 AR	CCAACAGATTGTCTTGTCATG
HEM3 BR	GTCTCTGGATGACCTTCCA
HEM3 CR	GACACCAAGATGATGAAGATTC

KAP123:

***Pta1: 1:**F2/R1, **2:**DN1/R7, **3:**DN2/R8, **4:**DN3/DN10, **5:**DN4/DN10, **6:**DN5/DN13*

***Rna15: 1:**F2/R1, **2:**DN1/R7, **3:**DN2/R8, **4:**DN3/DN10, **5:**DN4/DN10, **6:**DN5/DN13*

***Rat1:** 1:F2/R1, **2:**DN3/DN10, **3:**DN4/DN10, **4:**DN5/DN12, **5:**DN5/DN13, **6:**DN6/DN12*

***Ser2p: 1:**DN1/R7, **2:**DN4/DN11**,** **3:**DN5/DN11, **4:**DN5/DN12, **5:**DN6/DN12*

***Ser5p 5' End: 1:**F1/R3, **2:**F2/R3, **3:**F3/R1, **4:**F4/R1*

***Ser5p 3' End: 1:**DN3/DN10, **2:**DN4/DN11, **3:**DN5/DN11*

**Name**	**Sequence**
KAP123 F1	CTGTTCCAGCATTATTGG
KAP123 F2	GTGCTCTAGTTCCATTGG
KAP123 F3	TGCTAGTGGATGTGTGG
KAP123 F4	TTAGCAGCCGAAGATGAC
KAP123 R1	CCTTATGCTTCATCTCTTC
KAP123 R3	GTGCCTAAGATTGGGTGG
KAP123 R7	AATTGCGTCGTCAACATC
KAP123 R8	GTTCTGGGATATTCGCCG
KAP123 DN1	CACCCAATCTTAGGCAC
KAP123 DN2	GCCAATCTTGTATGTATGTG
KAP123 DN3	GTTGACGACGCAATTCG
KAP123 DN4	GCAAGTTCAGGTCATCTTG
KAP123 DN5	TTGACGATGTATAGTACATG
KAP123 DN6	TGCAAGTTACCTGCAGG
KAP123 DN10	CCTGCAGGTAACTTGC
KAP123 DN12	TTGAACTGTGGTCCCTG
KAP123 DN13	GCACTATACACTCGTTGG

SUR1:

***Pta1: 1:**A1/R1, **2:**A1/R2, **3:**F0.5/R1, **4:**DN1/R5, **5:**DN3/R1*

***Rna15: 1:**F2/A2, **2:**F0.5/R1, **3:**DN1/R5, **4:**DN2/R6, **5:**DN3/R7, **6:**DN6/DN11*

***Rat1: 1:**F2/A2, **2:**F0.5/R1, **3:**DN1/R5, **4:**DN2/R6, **5:**DN3/R7, **6:**DN6/DN11*

***Ser2p: 1:**F3/A2, 2:DN1/R5, **3:**DN2/R6, **4:**DN3/R7, **5:**DN5/DN11*

***Ser5p 5' End: 1:**A2F/AF1, **2:**F7/A2R, **3:**F3/A2R, **4:**F3/A3R, **5:**F1/A2R*

***Ser5p 3' End: 1:**F3/A2, **2:**DN1/R5, **3:**DN7/DN12*

Name	Sequence
SUR1 A1	GCTTACTACAAGATGCATAG
SUR1 A2	ATGTCTCGATCTACATCC
SUR1 F0.5	CCACATCTTATCCTGCG
SUR1 F1	GCTTACTACAAGATGCATAG
SUR1 F2	GCGCATACCTAAGAACG
SUR1 F3	CCCTTACATGACTATTATGG
SUR1 F7	CATAGAACGTGCCGATG
SUR1 R1	GCAGTATCACTGGTACTT
SUR1 R2	CCAGTGAGTACTTAGAAG
SUR1 R5	GTAGTATGTCGTAAGAAGC
SUR1 R6	ATGATTATGTTGATGCTGG
SUR1 R7	GTATAGAGGGTAATATCACAG
SUR1 DN1	CTCCAACAATTCATATCAGAC
SUR1 DN2	CTGAAGACCAGTCTTGTAC
SUR1 DN3	CTTCTTACGACATACTACTCC
SUR1 DN5	ATTACCCTCTATACTACGATATC
SUR1 DN6	GAATATGAACACTTCCTCAC
SUR1 DN7	CATCGCAACGGAGTAAG
SUR1 DN11	GCGGCTACTAGTCGAG
SUR1 A1F	GTTGTGTATTGACGATACTG
SUR1 A1R	ATCGATGTATACACCACC
SUR1 A2R	ACGCAGGATAAGATGTGG
SUR1 A3R	ACGCAGGATAAGATGTGG

S3 Supplementary Figure S1

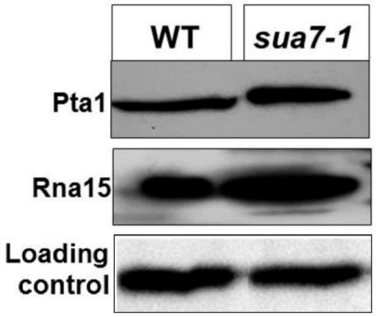

Supplementary Figure S1: Termination factor abundance is not reduced in sua7-1 mutant. *Western blot depicting the detection of termination factors Pta1 and Rna15, compared between wild-type and the sua7-1 mutant, along with loading control (actin).*

S4 Supplementary Figure S2

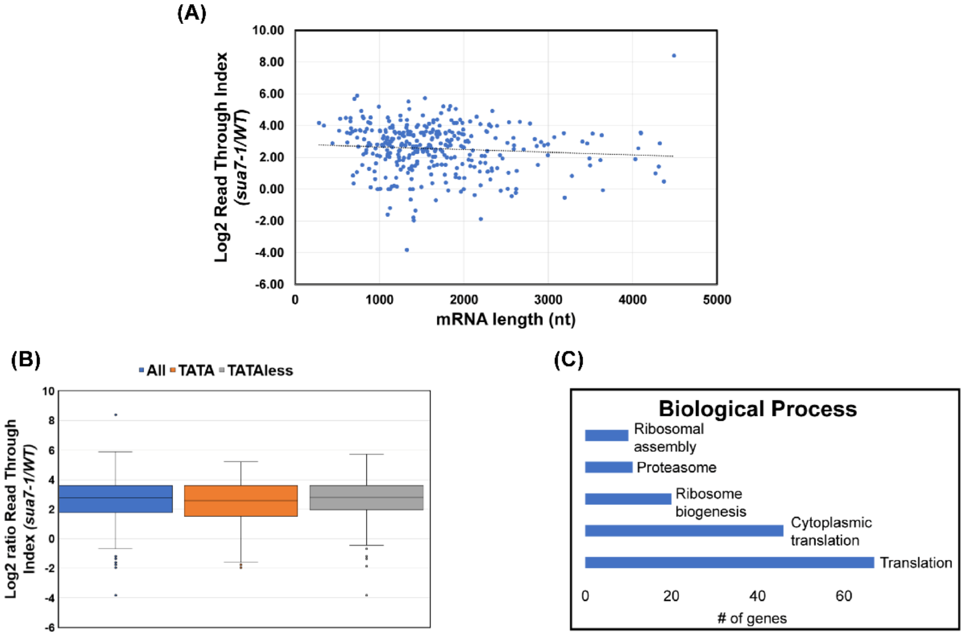

Supplementary Figure S2: TFIIB-dependent termination defect is not correlated to gene length, is independent of TATA box presence in promoter, and is pronounced in translation as well as ribosome function related genes. (A) *mRNA length is plotted on the x-axis and log2 RTI ratio of sua7-1/WT is on y=axis. A trendline is shown as a dotted line. Data is from three biological replicates.* ***(B)*** *The median log2 ratio of RTI for sua7-1/WT is compared across total genes analyzed (blue), genes with an associated TATA box (orange), and TATA-less genes (grey). Within the box and whisker plot, the middle line represents the median value of the corresponding RTI. The top and bottom lines represent the upper and lower quartiles. Data is from three biological replicates. Gene ontology analysis for total analyzed genes. The number of genes (x-axis) and their corresponding biological process (y-axis) are shown. Terms and analysis performed with NCBI DAVID gene ontology database.*

S5 Supplementary Figure S3

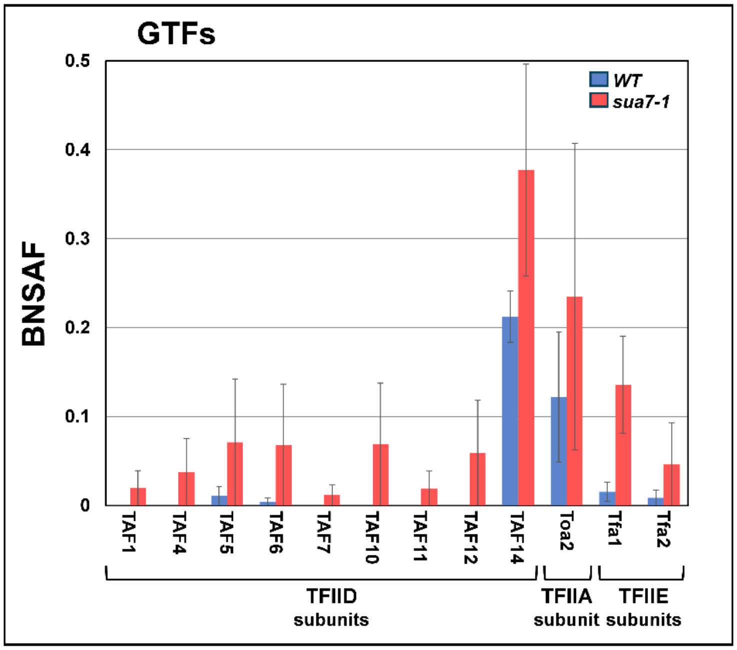

Supplementary Figure S3: TFIIB-general transcription factors interactions are not compromised in sua7-1 mutant. *BNSAF values of subunits of TFIID, TFIIA and TFIIE in affinity purified TFIIB from chromatin fraction in sua7-1 and WT cells. Data is form at least three biological replicates. p-values obtained from a standard, paired t-test. Error bars represent one unit of standard error based on four biological replicates.*
